# The value of right heart contrast echocardiography combined with migraine rating scale in evaluating the efficacy of patent foramen ovale closure

**DOI:** 10.1186/s12872-023-03411-8

**Published:** 2023-08-09

**Authors:** Yonghong Niu, Junxiang Pan, Shasha Fan, Lianyi Wang, Xiujie Tang

**Affiliations:** 1 Department of Cardiology, First Affiliated Hospital of Tsinghua University, Beijing, 100016 China; 2 Department of Pediatrics, First Affiliated Hospital of Tsinghua University, Beijing, 100016 China

**Keywords:** Right heart contrast echocardiography, Patent foramen ovale, Transcatheter closure, Visual analogue scale, Headache impact test-6, Migraine disability assessment questionnaire

## Abstract

**Objectives:**

To investigate the clinical values of right heart contrast transthoracic echocardiography (cTTE) combined with migraine rating scale in evaluating the efficacy of patent foramen ovale (PFO) closure.

**Methods:**

From January 2018 to December 2021, a total of 68 hospitalized patients, 21 males and 47 females, who were treated with transcatheter closure of PFO-induced migraine in the Heart Center of the First Affiliated Hospital of Tsinghua University were selected, with the age of 38.4 ± 11.9 years old. The changes of right heart contrast transthoracic echocardiography (cTTE), visual analogue pain score(VAS), headache impact test-6(HIT-6) and migraine disability assessment questionnaire(MIDAS) before and 6 months after PFO occlusion were compared.

**Results:**

Pre-operative cTTE data show that 36 patients (52.9%) had moderate right-to-left shunt (RLS), and 32 patients (47.1%) had massive RLS. cTTE was reexamined 6 months after operation and 1 case in the moderate RLS group had minimal RLS, 2 cases in the large RLS group had minimal RLS, and no shunts were seen for the rest. The VAS, HIT-6 and MIDAS scores before and 6 months after the operation were 7.65 ± 1.39 vs. 1.28 ± 1.53, 70.78 ± 6.82 vs. 41.53 ± 6.07, and 30.60 ± 13.24 vs. 1.93 ± 3.87, respectively. All the indexes 6 months after the operation significantly improved compared with the preoperative baseline (*P* < 0.05).

**Conclusions:**

cTTE combined with migraine evaluation scale could be used as an objective index to evaluate the clinical effect of PFO occlusion.

## Introduction

The foramen ovale is located at the junction of the primary atrial septum and the secondary atrial septum in the embryonic stage, and it is usually closed within 1 year after birth. Patent foramen ovale (PFO) is the consequence of failed closure of the foramen ovale, with approximately 25% prevalence in the adult population worldwide [1]. Del Sette et al. [2] first proposed that migraine was linked to PFO in 1998. In recent years, more and more studies have described the relevance between migraine and PFO [3]. The mechanism of migraine caused by PFO is mainly related to the abnormal right to left shunt (RLS), resulting in that substances that should enter the pulmonary circulation under normal conditions directly enter the systemic circulation, causing abnormal contraction and relaxation of blood vessels in the brain [4]. The application of transthoracic echocardiography (TTE) and transesophageal echocardiography (TEE) has greatly improved the accuracy of the diagnosis of PFO, but the sensitivity of RLS examination is relatively low. It has been confirmed that contrast transthoracic echocardiography (cTTE) can dynamically display the presence or absence of RLS in PFO in real-time, and can quantitatively assess the flow volume of RLS [5]. However, whether the RLS results displayed by cTTE are associated with the severity of migraine and its value in the follow-up of interventional occlusion needs to be further studied.

In this study, we conducted a self-controlled, retrospective analysis to evaluate clinical data of patients with PFO-caused migraine who received transcatheter closure procedures. We compared the changes of cTTE, VAS score, HIT-6 score, and MIDAS score before and 6 months after the operation, and explored the clinical value of cTTE in combination with migraine assessment scale in the evaluation of the efficacy of transcatheter closure.

## Materials and methods

### Subjects

68 patients diagnosed with PFO-caused migraine who further received transcatheter closure from January 2018 to December 2021 at the Heart Center of the First Affiliated Hospital of Tsinghua University (Beijing Huaxin Hospital) were selected, including 21 males (30.8%) and 47 females (69.1%), with the age of (38.4 ± 11.9) years old, BMI:23.48 ± 3.58 kg/m^2^.They were complicated with hypertension (10 patients), diabetes mellitus (5 patients), hyperlipidemia (4 patients), cervical spondylosis (2 patients), and hyperuricemia disease (2 patients). No specific anti-migraine medications were used before surgery, while aspirin 100 mg/day in combination with clopidogrel 75 mg/day were administered for 6 months after the procedure.

The inclusion criteria were as follows: (1) Patients met diagnostic criteria for migraine according to the third edition of International Society for Headache (ICHD-III R1) in 2013 [6], and there were no clear causes of recurrent headaches or ineffective drug treatment; (2) Diagnosed PFO through TTE/TEE examination; (3)Positive cTTE, moderate or larger RLS volume. All patients have signed informed consent forms. Exclusion criteria: (1) Migraine caused by intracranial space-occupying lesions, cerebrovascular malformations, cerebrovascular stenosis, etc.;(2) Contraindications for transcatheter PFO closure (contraindications for the use of antiplatelet drugs, thrombosis of the inferior vena cava or pelvic vein, etc.);(3) Clinical contraindications of cTTE (severe cyanosis with large intracardiac shunt flow, severe pulmonary hypertension, severe emphysema, respiratory insufficiency, severe anemia, acidosis, severe cardiac and renal insufficiency, acute coronary syndrome, etc.) [7].

#### Ethics approval and consent to participate

The study followed the principles of the Declaration of Helsinki and was approved by the clinical research ethics committee of Beijing Huaxin Hospital (2023–19). All participants in the study provided Informed consent after being fully informed of the purpose of the research.

### Echocardiography

All participants were diagnosed with PFO through TTE/TEE examination preoperatively. cTTE examination was conducted preoperatively and 6 months after operation. Images were interpreted by two chief echocardiologists in a double-blind method. Morphology and size of foramen ovale as well as blood flow signals were evaluated.

#### TTE/TEE

Both TTE and TEE were performed with Siemens SC 2000 ultrasonic diagnostic system. TTE probe frequency: 1.25–4.5 MHz. Multiple views including the aortic short-axis view and subxiphoid two atria were selected to fully display and observe the shape of the atrial septum. Color doppler flow imaging (CDFI) was used to observe the atrial horizontal shunt. TEE probe frequency: 3.0–6.3 MHz. TEE probe was inserted into the middle esophagus, and the horizontal view of both atria was obtained. The horizontal structure of the foramen ovale was observed. The probe was adjusted to 80° ~ 100° to fully display the entrance views of the superior and inferior vena cava, and the longitudinal structure of the foramen ovale was examined in a clockwise direction from 0° ~ 180°. CDFI was combined to display the atrial horizontal shunt.

#### cTTE

##### cTTE was performed in the standard apical 4-chamber view. The agitated saline contrast

(8ml normal saline + 1 ml air + 1 ml blood pumped back) was administrated through the left median cubital vein. Patients quickly released and expired (Valsalva maneuver) when the microbubbles entered the right atrium. The presence of PFO is presumed 3–5 cardiac cycles after complete opacification of the right atrium. The presence of the left atrium bubbles was observed. The shunt grading of PFO is classified as grade 0 (no microbubble), grade I (1–9 microbubbles/frame), grade II (10–30 microbubbles/frame), and grade III (> 30 microbubbles/frame), wherein grade I is defined as small shunt, grade II is defined as moderate shunt, and grades III and IV are defined as large shunt [7].

#### PFO operation

The patients were positioned supine and anesthetized with endotracheal intubation. Sterile draping was applied following routine disinfection, and the right femoral vein was punctured. Heparin was administered at a dose of 100U/kg. Under the guidance of transesophageal echocardiography, an ultra-slip guidewire was inserted, followed by the introduction of a sheath. The occlusion device delivery system was then exchanged, and an occluder of appropriate size was successfully deployed to close the oval foramen, according to the size and shape of the defect. Transesophageal echocardiography confirmed the absence of shunting at the atrial level and that all valve functions were unaffected, the sheath was removed, and after local compression dressing, the patient was safely returned to the ward.

#### Assessment of migraine

VAS, HIT-6 and MIDAS were performed preoperatively and 6 months postoperatively to assess the severity of migraine symptoms and the relief effect. The above scoring scales were all guided by professionals. The requirements were stated before patients filled out the forms by themselves. For low-educational level patients who were unable to complete the forms, the investigator would explain according to the requirements, then patients themselves would make the judgments to fill out the forms with the investigator’s help. The questionnaire was completed and checked on the spot. The investigation was supplemented in time.

#### Visual analogue scale (VAS)

The subjective headache severity was quantified using a straight horizontal measuring scale with 10 cm of fixed length and 10 scales marked. A score of 0 indicated no pain, and a score of 10 indicated the worst pain. The classification criteria were mild pain (1–3 points), moderate pain (4–6 points), and severe pain (7–10 points) [8].

#### Headache Impact Test -6 (HIT-6)

A HIT-6 questionnaire was used for evaluating the disability of headache, regarding the frequency of headache onset, the frequency of headache affecting daily life, and the frequency of headache affecting emotions. The questionnaire consists of 6 questions, each of which has five answers: "Never, rarely, sometimes, very often, always", which were recorded as 6 points, 8 points, 10 points, 11 points and 13 points. The total HIT-6 score was calculated by summing up each score. Based on the obtained HIT-6 score(range 36–78 points), the disability was quantified using the following 4 impact grades: no or mild impact (36–49 points), moderate impact (50–55 points), significant impact (56–59 points) and substantial impact (60–78 points) [9].

#### Migraine Disability Assessment Questionnaire (MIDAS)

MIDAS score was calculated by scoring either days of missed activity or days where productivity was reduced by at least half due to migraine in the past 3 months. Five questions are asked regarding the lost time in three domains: school work or work for pay; household work or chores; and family, social, and leisure activities. Based on the MIDAS score, the severity of headache was graded as: little or no disability (0–5 points), mild disability (6–10 points), moderate disability (11–20 points) and severe disability (≥ 21 points) [10].

### Statistical analysis

Statistical analysis was performed using SPSS 20.0. The categorical variables were summarized using percentages.The continuous data with normal distribution were summarized as mean ± standard deviation values and compared using Student’s t-test. A two-sided *p*-value < 0.05 was considered statistically significant.

## Results

From January 2018 to December 2021, a total of 68 hospitalized patients received transcatheter closure due to PFO-caused migraine at the Heart Center of the First Affiliated Hospital of Tsinghua University.

**Fig. 1:**
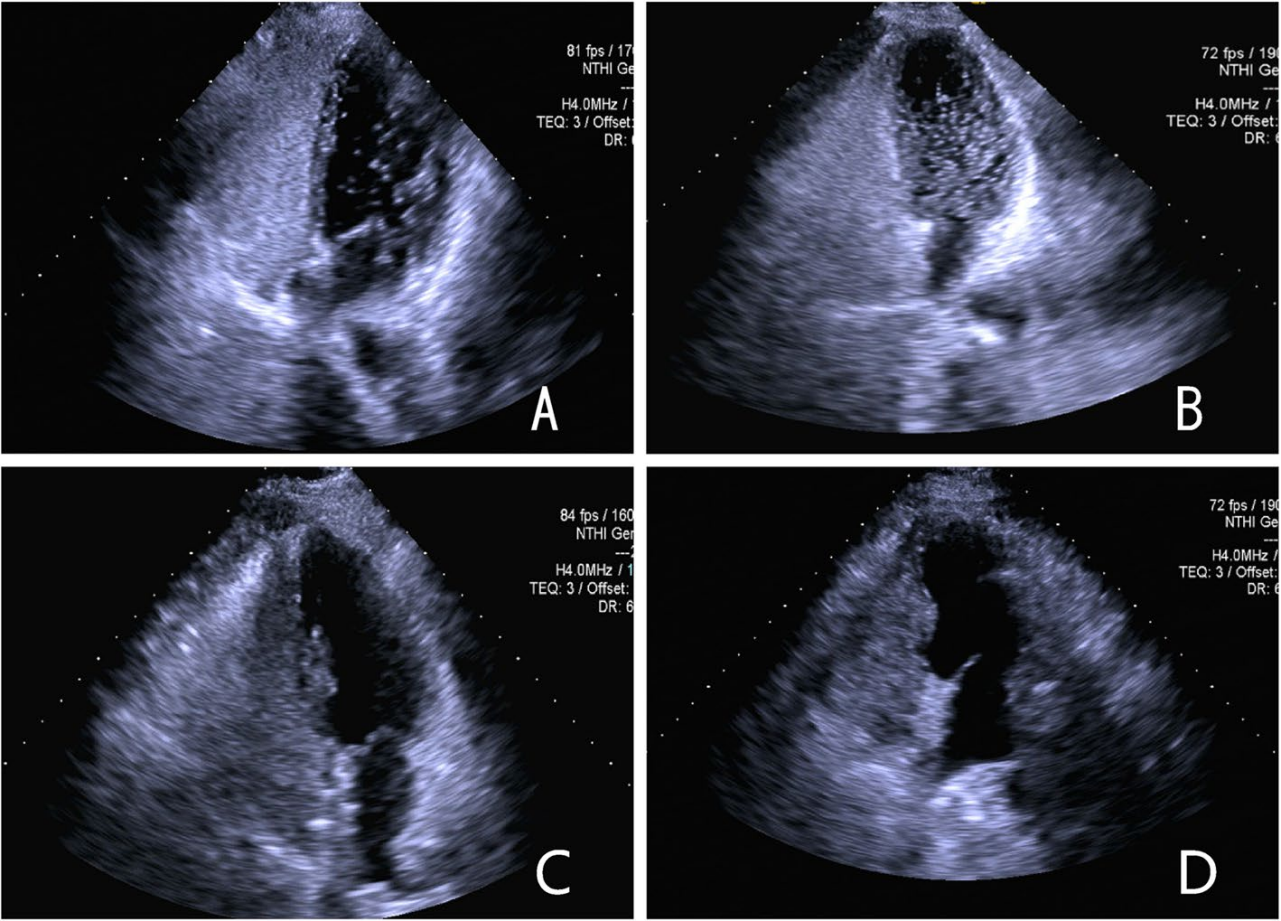
Results of contrast echocardiography of right heart

Preoperative cTTE data showed that 36 patients (52.9%) had moderate right-to-left shunt (RLS) (Fig. 1A), and 32 patients (47.1%) had large RLS (Fig. 1B). A follow-up of cTTE at 6 months postoperatively revealed 1 case of minimal RLS in the moderate RLS group, 2 cases of minimal RLS in the large RLS group (Fig. 1C), and no shunts in the remainder (Fig. 1D).

**Fig. 2:**
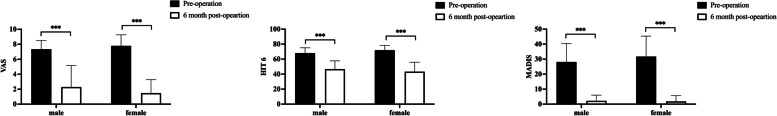
Comparison of VAS, HIT-6 score and MIDAS score before and 6 months after PFO closure in different gender (‾x ± s)

**Fig. 3:**
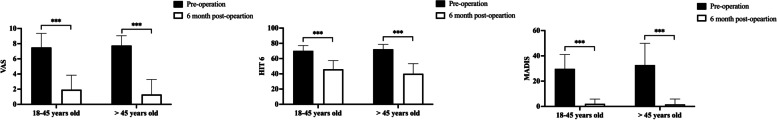
Comparison of VAS, HIT-6 score and MIDAS score before and 6 months after PFO closure in different age groups (‾x ± s)

Preoperative: (1)VAS score was (7.65 ± 1.39), 16 cases (23.53%) had moderate pain, 52 cases (76.47%) had severe pain; (2) The HIT-6 score was (70.78 ± 6.82), of which, 1 case (1.47%) was considered as significant impact, and 67 cases (98.53%) as substantial impact; (3) The MIDAS score was (30.60 ± 13.24), of which, 1 case (1.47%) was little disabled, 1 case (1.47%) was mild disabled, 14 cases (20.59%) were moderately disabled, and 52 cases (76.47%) were severely disabled. 6 months postoperative: (1) VAS score was (1.28 ± 1.53) points, 35 cases (51.47%) had no pain, 30 cases (44.12%) had mild pain, and 3 cases (4.41%) had moderate pain; (2) The HIT-6 score was (41.53 ± 6.07), among which 65 cases (95.59%) had no or mild impact, and 3 cases (4.41%) had moderate impact. (3) The MIDAS score was (1.93 ± 3.87), including 5 cases (7.35%) of mild disability, 3 cases (4.41%) of moderate disability, and no case of severe disability, and the remaining 60 cases (88.24%) of no disability. All the scores significantly improved after 6 months compared with the preoperative baseline level (*P* < 0.05) (Table 1).The relationship between gender and stunt severity is shown in Table 2 and Fig. 2.The relationship between age and stunt severity is shown in Table 3 and Fig. 3.

**Table 1:** Comparison of VAS, HIT-6 score and MIDAS score before and 6 months after PFO closure (‾x ± s)

Indicators	Before operation	Six months post-op	∆Before operation-Six months post-op	T	P
VAS	7.65 ± 1.39	1.28 ± 1.53	6.37士2.08	25.25	6.02 × 10^–36^
HIT-6	70.78 ± 6.82	41.53 ± 6.07	29.25士8.90	27.09	8.09 × 10^–38^
MIDAS	30.60 ± 13.24	1.93 ± 3.87	28.68士12.41	19.06	9.18 × 10^–29^

**Table 2:** Comparison of VAS, HIT-6 score and MIDAS score before and 6 months after PFO closure in different gender (‾x ± s)

male				
Scores	Pre-operation	6 months post-operation	T value	*P* value
VAS score	7.33 ± 1.15	2.29 ± 2.88	7.66	1.51 × 10^–7^
HIT-6 score	68.10 ± 7.11	46.67 ± 11.06	7.22	5.38 × 10^–7^
MIDAS score	28.20 ± 12.30	2.19 ± 3.84	11.23	4.33 × 10^–10^
female				
Scores	Pre-operation	6 months post-operation	T value	*P* value
VAS score	7.78 ± 1.47	1.47 ± 1.82	17.74	3.33 × 10^–22^
HIT-6 score	71.98 ± 6.40	43.25 ± 12.50	15.16	1.65 × 10^–19^
MIDAS score	31.72 ± 13.62	1.81 ± 3.92	15.71	4.17 × 10^–20^

**Table 3:** Comparison of VAS, HIT-6 score and MIDAS score before and 6 months after PFO closure in different age (‾x ± s)

18–45 years old						
Scores	Pre-operation		6 months post-operation	T value		*P* value
VAS score	7.50 ± 1.94		1.94 ± 1.91	14.29		9.99 × 10^–19^
HIT-6 score	70.17 ± 6.97		46.06 ± 11.27	12.95		4.11 × 10^–17^
MIDAS score	29.73 ± 11.28		2.08 ± 3.71	18.26		5.56 × 10^–23^
> 45 years old						
Scores	Pre-operation	6 months post-operation			T value	*P* value
VAS score	7.67 ± 1.32	1.24 ± 1.95			12.50	2.83 × 10^–10^
HIT-6 score	69.10 ± 17.78	38.19 ± 15.59			9.84	1.90 × 10^–7^
MIDAS score	31.43 ± 12.30	1.48 ± 4.20			8.22	2.04 × 10^–9^

## Discussion

Migraine is a common chronic disease characterized by recurrent unilateral pulsatile headache, which often has a serious impact on patients' daily life and work. However, the pathogenesis of migraine is complicated. In recent years, studies have shown that the prevalence of PFO in migraine patients is significantly higher than that of the normal population [3]. The underlying pathophysiology of PFO-caused migraine is currently considered as follows: (1) Microembolic hypothesis: venous paradoxical embolisms; (2) Vasoactive substance hypothesis: 5- hydroxytryptamine and other substances are not cleared by lung circulation, and these chemicals or microthrombus can lead to transient neurological dysfunction [4]. When Wilmshurst et al. [11] performed PFO closure to prevent decompression sickness/stroke in 2000, they found by chance that it could reduce the symptoms of migraine. Since then, Transcatheter closure of PFO has been used as one of the means for the treatment of migraine,but Andrew Dowson et al. found the clinical efficacy of PFO closure as migraine treatment is yet unclear [12]. So we investigate the clinical values of right heart contrast echocardiography (cTTE) combined with migraine rating scale in evaluating the efficacy of PFO closure.

The diagnosis of PFO is mainly based on echocardiography. TTE scanning has a low resolution for the evaluation of atrial septal shunt due to the interference of subcutaneous tissues and lung gas, which makes it difficult to accurately diagnose and measure PFO, and missed diagnoses were often seen with this method. TEE is considered the gold standard for PFO diagnosis. However, oral mucosal anesthetics are required for TEE procedure. During TEE examination, patients' pharynx is easily irritated by physical stimulation, resulting in nausea and vomiting. It is difficult to complete the Valsalva maneuver with TEE. Therefore, TEE is limited for follow-up after PFO closure. Combined with the Valsalva maneuver, cTTE examination can be used to assess the closure of the foramen ovale by performing contrast imaging of the right heart, to observe whether any microbubbles enter the left atrium through the unclosed foramen ovale after the full opacification of right atrium. It can not only display the structure of the foramen ovale but also quantify RLS by observing the number of microbubbles in the left atrium [5]. However, whether the RLS result displayed by cTTE is related to the severity of migraine, as well as the value of cTTE in the follow-up of interventional occlusion needs to be further studied.

Evaluating the severity of migraine not only helps to understand the impacts of migraine on patients' physical, psychological and social life but also helps to choose the effective treatment and follow-up strategy. Several methods have been used to assess the severity of migraine. The specific assessment tool used in clinical depends on the specific medical needs. In this study, VAS score, HIT-6 score and MIDAS score were used to assess the severity of migraine and disability of patients, respectively. VAS score is a simple and effective tool for measuring migraine intensity [8]. HIT-6 score is used to generate quantitative and pertinent information on the impact of headaches on patients’ daily life, which covers six aspects such as pain, social functioning, cognitive functioning, role functioning, psychological distress, and vitality [9]. MIDAS score is a tool to measure headache-related disability quantitatively. The above three scoring tools have been widely used to evaluate and observe the impact of migraine [13–15].

In this study, we conducted a self-controlled, retrospective analysis to evaluate clinical data of patients with PFO-caused migraine who received transcatheter closure procedures. By comparing the changes of cTTE, VAS, HIT-6 score and MIDAS score before and 6 months after the procedure, we explored the clinical value of cTTE in combination with the migraine assessment scale in the evaluation of the efficacy of PFO closure. In this study, the VAS scores indicated moderate and severe pain in all patients, 98.53% of patients’ HIT-6 score was considered as severe impact, and 97.06% of patients’ MIDAS score was considered as moderate and severe disability. The cTTE examination indicated that all the patients had RLS of moderate or above. After transcatheter closure of the PFO, cTTE revealed RLS in only three patients. Compared with those before treatment, VAS, HIT-6 and MIDAS scores after transcatheter closure significantly decreased, and the frequency, duration and severity of migraine symptoms in patients were significantly improved, suggesting that transcatheter closure could significantly improve PFO-caused migraine. In terms of age, the HIT6 scores for the age group of 18–45 years were significantly higher than those for the age group of over 45 years. However, no significant age differences were observed in the VAS and MIDAS scores. Notably, regardless of age group, postoperative scores showed a marked improvement compared to preoperative ones. As for gender, no significant differences were found in the scores. Furthermore, irrespective of gender, there was a noticeable improvement in postoperative scores compared to those before the operation.

The analysis showed that PFO was the pathological basis for RLS. When RLS disappeared after mechanical occlusion, the symptoms of migraine were also relieved and disappeared. Six months after the operation, cTTE showed that only three patients had mild RLS. Generally, complete endothelization of occluder happens within half a year after implantation [16]. In theory, a better degree of occluder endothelization can be achieved with a longer implantation time, resulting in further improved migraine symptoms.

In summary, interventional occlusion treatment can effectively alleviate migraine caused by PFO and is worthy of clinical promotion. As a non-invasive examination, cTTE is easy to carry out in clinical practice to accurately assess the efficacy of transcatheter PFO closure by combining with VAS, HIT-6 and MIDAS scores. These evaluation methods can also be used for postoperative follow-up. Due to the limited sample size of this study, it is impossible to rule out the bias caused by other factors. A large-sample, multi-center, randomized, double-blind controlled study is needed to obtain more powerful evidence to support the clinical value of the methods above performed before and after PFO occlusion.

## Data Availability

The datasets regarding the current study are available from the corresponding author upon reasonable request.
